# Eco-Friendly Fluorescent ELISA Based on Bifunctional Phage for Ultrasensitive Detection of Ochratoxin A in Corn

**DOI:** 10.3390/foods10102429

**Published:** 2021-10-13

**Authors:** Weipeng Tong, Hao Fang, Hanpeng Xiong, Daixian Wei, Yuankui Leng, Xinyu Hu, Xiaolin Huang, Yonghua Xiong

**Affiliations:** 1 State Key Laboratory of Food Science and Technology, Nanchang University, Nanchang 330047, China; 402337519017@email.ncu.edu.cn (W.T.); 357900210001@email.ncu.edu.cn (H.F.); 402337520004@email.ncu.edu.cn (H.X.); 412314919055@email.ncu.edu.cn (D.W.); xiaolin.huang@ncu.edu.cn (X.H.); yhxiongchen@163.com (Y.X.); 2 School of Food Science and Technology, Nanchang University, Nanchang 330047, China; 3 School of Qianhu, Nanchang University, Nanchang 330031, China; 7901119026@email.ncu.edu.cn; 4 Jiangxi-OAI Joint Research Institute, Nanchang University, Nanchang 330047, China

**Keywords:** M13 bacteriophage, enzyme container, mercaptopropionic acid-modified quantum dot, Ochratoxin A, fluorescent immunoassay

## Abstract

Conventional enzyme-linked immunosorbent assay (ELISA) is commonly used for Ochratoxin A (OTA) screening, but it is limited by low sensitivity and harmful competing antigens of enzyme-OTA conjugates. Herein, a bifunctional M13 bacteriophage with OTA mimotopes fused on the p3 protein and biotin modified on major p8 proteins was introduced as an eco-friendly competing antigen and enzyme container for enhanced sensitivity. Mercaptopropionic acid-modified quantum dots (MPA-QDs), which are extremely sensitive to hydrogen peroxide, were chosen as fluorescent signal transducers that could manifest glucose oxidase-induced fluorescence quenching in the presence of glucose. On these bases, a highly sensitive and eco-friendly fluorescent immunoassay for OTA sensing was developed. Under optimized conditions, the proposed method demonstrates a good linear detection of OTA from 4.8 to 625 pg/mL and a limit of detection (LOD) of 5.39 pg/mL. The LOD is approximately 26-fold lower than that of a conventional horse radish peroxidase (HRP) based ELISA and six-fold lower than that of a GOx-OTA conjugate-based fluorescent ELISA. The proposed method also shows great specificity and accepted accuracy for analyzing OTA in real corn samples. The detection results are highly consistent with those obtained using the ultra-performance liquid chromatography-fluorescence detection method, indicating the high reliability of the proposed method for OTA detection. In conclusion, the proposed method is an excellent OTA screening platform over a conventional ELISA and can be easily extended for sensing other analytes by altering specific mimic peptide sequences in phages.

## 1. Introduction

Ochratoxin A (OTA) is a *Penicillium*- and *Aspergillus*-derived mycotoxin [1] with high nephrotoxicity, hepatotoxicity, immunotoxicity, teratogenicity, carcinogenicity and mutagenicity [2]. The widely occurring OTA contamination in various crops (e.g., corn, wheats, beans, coffee and cocoa) poses a serious human health threat and huge economic loss worldwide [3]. As such, many countries and international organizations have set up maximum residue levels (MRLs) of OTA, which ranges from 0.5 to 10 μg/kg in different food commodities according to the European Union standards [4]. To prevent contaminated foods or feedstuffs moving into the food chain, various methods have been adopted to analyze OTA, such as high-performance liquid chromatography (HPLC) [5], liquid chromatography–mass spectrometry [6], an immunochromatographic strip test [7] and an enzyme-linked immunosorbent assay (ELISA) [8]. Among them, an ELISA is the most popular for the high-throughput screening of OTA-polluted samples because of its high inspection speed, low cost and excellent specificity [9,10]. However, a conventional ELISA suffers from limited sensitivity because of the low signal intensity of enzymatic reaction-derived chromogenic products [11,12]. Moreover, using enzyme–OTA conjugates as competing antigens leads to a serious secondary pollution and poses a noticeable occupational hazard [13] because of the consumption of toxic OTA and toxic organic reagents for its preparation. Hence, competing immunoassays with enhanced sensitivity and eco-friendliness for OTA screening should be developed.

The utilization of fluorescent signals as replacements of colorimetric signals is a simple and promising method to increase the sensitivity of ELISA [14,15]. Highly sensitive fluorescent immunoassays involving various fluorescent compounds, including organic dyes, upconverting nanoparticles (UCNPs) and quantum dots (QDs) have been developed. However, traditional organic fluorescent dyes experience high background noise from autofluorescence and are susceptible to photobleaching [16] and UCNPs possess a relatively low fluorescence intensity. By comparison, QDs are optimal fluorescent reporters because of their broad excitation, narrow emission spectra, large Stokes shifts and high photostability [17,18]. Recently, immunoassays based on the fluorescence switching of the quenching of mercaptopropionic acid-modified QDs (MPA-QDs) induced by hydrogen peroxide (H_2_O_2_) have been widely explored because of the simple and sensitive signal transduction strategy for QDs without a conjugation process [19,20,21]. The bioconjugation-free application of QDs also results in the high robustness of fluorescent signals. In this type of immunoassays, glucose oxidase (GOx) and catalase that can regulate the H_2_O_2_ concentration are used as labels.

The use of traditional competing antigens of enzyme–OTA conjugates limits the detection sensitivity because one OTA molecule can only competitively inhibit the binding of one enzyme molecule. It also leads to severe secondary pollution due to the consumption of toxic OTA and other toxic organic reagents. Hence, new types of competing antigens, especially those that are loaded with enhanced amounts of enzymes and exhibiting eco-friendliness, should be explored. For example, M13 bacteriophage (M13 phage) displaying a mimic antigen epitope and loaded with enzymes has been regarded as a promising alternative competing antigen. It is a 900-nanometer-long and 6.5-nanometer-thick filamentous virus [22] that is noninfectious to humans and composed of 2700 copies of the major p8 protein and 3–5 copies of minor p3, p6, p7 and p9 proteins at both ends [23,24]. It can be easily modified so that it can mimic small molecules or protein compounds by integrating mimotopes or ligands on the N-terminal of p3 protein via a gene modification–fusion expression process [25,26]. In addition, the major p8 proteins of M13 can be extensively functionalized as a container for the loading of signal transducers or regulators (e.g., gold nanoparticles, fluorescent dyes and enzymes) because of their abundant copies on phage particles; consequently, the signal intensities of biosensors can be remarkably enhanced [27,28]. For instance, Lee et al. [29] adopted the M13 phage as a biological template to amplify the surface-enhanced Raman scattering signal for protein sensing. Fang et al. [30] used a sulfydryl-modified M13 phage as an Au-Ag nanozyme container to improve the sensitivity of a colorimetric biosensor.

In a previous report, an OTA mimicking M13 phage integrated with an OTA mimotope at the site of the p3 proteins, termed as M13_OTA_, is panned from a commercial phage display random seven peptide library by using an anti-OTA monoclonal antibody as a receptor [31]. Herein, an eco-friendly fluorescent ELISA (FLISA) for the highly sensitive detection of OTA in real corn was proposed, wherein a biotinylated M13_OTA_ phage was applied as a competing antigen and container of the GOx enzyme, and MPA-QDs were used as signal transducers. The detection performance, limit of detection (LOD), 50% competitive inhibition concentration (IC_50_), accuracy and reliability of the proposed M13_OTA_-FLISA were assessed and compared with those of a conventional HRP-based ELISA.

## 2. Materials and Methods

### 2.1. Materials and Apparatus

The following materials were used: OTA, deoxynivalenol (DON), aflatoxin B1 (AFB1), citrinin (CIT), fumonisin B1 (FB1), zearalenone (ZEN), Gox and bovine serum albumin (BSA; Sigma-Aldrich Chemical Co., St. Louis, MO, USA); streptavidin and streptavidin-FITC (Solarbio, Beijing, China); anti-OTA ascitic fluids (Wuxi Zodoboer Biotech, Co., Ltd., Wuxi, China); sulfosuccinimidyl 6-(biotinamido)hexanoate (Macklin, Shanghai, China); MPA-modified CdTe QDs (Technical Institute of Physics and Chemistry, CAS); 96-well plates (Corning Inc., New York, NY, USA); and M13 bacteriophage with a peptide sequence of GMSWMMA (M13_OTA_), which exhibits the highest sensitivity to OTA detection (Prof. Xuelan Chen’s lab). Pure water was prepared using a Milli-Q system (Millipore, Milford, MA, USA). All the other chemicals (Sinopharm Chemical Corp., Shanghai, China) were of analytical grade and applied without further purification.

### 2.2. Optimization of GOx-Mediated Fluorescence Quenching of MPA-QDs

Two key parameters including the pH value and concentration of MPA-QDs were optimized by three experiments to enable the highest sensitivity of MPA-QDs solution for GOx in the presence of glucose. In the first experiment, 50 μL of 25 nM MPA-QDs in phosphate buffered (PB, pH = 5.0~8.0) was added to 100 μL of PB with the same pH, and then the emission at 590 nm (Em_590_) was recorded using a multimode microplate reader (Thermo Varioskan Flash, Thermo, Waltham, MA, USA) under a 310-nanometer excitation to assess the effect of pH on the fluorescence of MPA-QD. In the second experiment, 50 μL of MPA-QDs (12.5–33 nM in PB, pH = 7.5) was added to 100 μL of H_2_O_2_ solution (0~100 μM in PB buffer, pH = 7.5), and the fluorescence signal (Em_590_) was recorded 15 min later to evaluate the effect of QD concentration on the sensitivity for H_2_O_2_. Finally, to obtain an optimized pH value, assays below were conducted in PB buffer with different pH values. First, 50 μL of GOx solution (1.0 pg/mL-10 μg/mL) was incubated with 50 μL of glucose solution (1 mg/mL) for 1 h, then 50 μL of MPA-QDs (optimal concentration obtained from the second experiment) was added, Em_590_ was recorded 15 min later. Thereafter, standard curves were established to assess the sensitivity for GOx by plotting the fluorescence quenching efficiency (1-F/F_0_) against GOx concentration, where F and F_0_ were resultant Em_590_ values with or without GOx.

### 2.3. Propagation of M13_OTA_ Bacteriophage

*Escherichia coli* ER2738 cells were grown in a Luria-Bertani (LB) medium containing 10 μg/mL of tetracycline at 37 °C for 8 h. Then, 200 μL of the above E. coli cells and 1 μL of M13_OTA_ phage (10^13^ pfu/mL) were inoculated into 20 mL of LB liquid medium and cultured at 37 °C for 4.5 h in an incubator while shaking at 250 rpm to propagate M13_OTA_ bacteriophages. After the cell debris were removed by spinning at 5000 rpm for 10 min, M13_OTA_ phages were precipitated at 4 °C overnight by adding 5 mL of a PEG-NaCl solution (2.5 M NaCl, 20% PEG-8000) to 20 mL of the supernatant. Precipitates were collected via centrifugation (13,500 rpm, 10 min) and resuspended in 1 mL of PB solution (pH = 7). The cell debris were removed by spinning at 5000 rpm for 10 min. Afterward, 200 μL of PEG-NaCl solution was added to the supernatant and incubated on ice for 1 h. The M13_OTA_ phage was obtained by spinning at 13,500 rpm for 10 min and re-suspended with 200 μL of PB buffer (pH = 7).

The concentration of the amplified M13_OTA_ phage was determined via the plate count method [30]. Briefly, a swatch of ER2738 cells grown on an LB/Tet plate (LB plate containing 10 μg/mL of tetracycline) was inoculated into 5–10 mL of LB medium and incubated under shaking for 6-10 h to mid-log phase (OD600 = 0.5). Then, 10 μL of the phage solution (10 to 10^9^-fold diluted) was used to infect the ER2738 cells (200 μL). After a 5-minute incubation at room temperature, the mixture was added into 3 mL of the top agar, and the culture was immediately poured onto a pre-warmed LB/IPTG/X-gal plate. The plates were cooled at room temperature for 5 min, then inverted and incubated overnight at 37 °C. Thereafter, the plaques on each plate were counted to address the titer of the amplified M13_OTA_ phage. Plaques counting results (Figure S1) showed 34 plaques from 10 μL of 10^8^-fold dilution in the infected cells, indicating that the stock titer of phage solution was 3.4 × 10^12^ pfu/mL.

Solutions and plates used above were prepared according to the following recipes. LB medium was prepared by dissolving 10 g of Bacto-Tryptone, 5 g of yeast extract, 5 g of NaCl in 1 L of pure water. IPTG/X-gal Stock was prepared by mixing 1.25 g of isopropyl-β-D-thioglactoside (IPTG) and 1 g of 5-bromo-4-chloro-3-indoyl-β-D-glactoside (X-gal) in 25 mL of dimethyl formamide (DMF). First, 1 mL of IPTG/Xgal was mixed with 1 L of autoclaved LB medium containing 15 g of agar, and then the medium was poured into the plates to obtain LB/IPTG/X-gal Plates. TOP Agar is LB Medium containing Bacto-Agar (7 g in 1 L of medium).

### 2.4. Preparation of Biotinylated M13_OTA_ Phage

Biotinylated M13_OTA_ phages were obtained by coupling sulfosuccinimidyl 6-(biotinamido) hexanoate (NHS-biotin) with the amine group of capsid proteins, especially the major p8 proteins, via the active ester method. Briefly, a certain amount of NHS-biotin was added into 1 mL of M13_OTA_ phage solution (1.2 × 10^12^ pfu/mL, pH 8.6), and incubated on ice for 4 h under vigorous stirring. Then, 200 μL of PEG-NaCl solution was added to the above mixture and incubated on ice for 1 h. A biotinylated M13_OTA_ phage was obtained by spinning at 13,500 rpm for 10 min, resuspended in 1 mL of PB buffer, and stored at 4 °C.

The dosage ratio of M13_OTA_ phage to NHS-biotin was optimized to maximize the loading capacity of biotinylated M13_OTA_ phage. Briefly, immunoassays described in Section 2.6 were conducted without OTA samples added based on biotinylated M13_OTA_ phages prepared from different dosage ratios of M13_OTA_ phage to NHS-biotin, and the fluorescence intensity of CdTe QDs were detected to evaluate the performance of biotinylated M13_OTA_ phages. Wherein, the concentrations of anti-OTA ascitic fluids and biotinylated M13_OTA_ phage were 1.0 μg/mL and 2 × 10^10^ pfu/mL, respectively.

### 2.5. Preparation of Biotinylated GOx (Biotin-GOx)

Biotinylated GOx was obtained by coupling the NHS-biotin with the amine group of GOx. Briefly, 36 μL of NHS-biotin (1 mg/mL) was added to 0.5 mL of GOx solution (2 mg/mL, pH 7.4) and reacted on ice for 4 h under vigorous stirring. The conjugates were dialyzed in phosphate buffered saline (PBS) solution (0.01 M, pH 7.4) for 72 h by using a dialysis bag. The final solution was stored in a refrigerator at −20 °C.

### 2.6. Procedure of M13_OTA_-FLISA for OTA Detection

A FLISA was developed by using biotinylated M13_OTA_ as a competing antigen and MPA-QDs as signal transducers. Briefly, 100 μL of protein G (25 μL/mL, diluted in 0.01 M PBS 8.6) was added to microplates and incubated overnight at 4 °C. After microplates were washed thrice with PBST (PBS buffer containing 0.05% Tween 20) and once with PBS, 100 μL of anti-OTA ascitic fluids (diluted in PBS, pH = 7.4) was added to each well and incubated at 37 °C for 1 h. After blocking with 50 mg/mL of BSA solution (pH = 7.4) for 2 h at 37 °C, the microplates were washed thrice with PBST and once with PBS. Subsequently, 50 μL of biotinylated M13_OTA_ phage (diluted in PBS) and 50 μL of the sample solution were added to each well and incubated at 37 °C for 1 h. After washing thrice with PBST and once with PBS, 100 μL of streptavidin solution (1 μg/mL) was added for 0.5 h of reaction at 37 °C. Subsequently, 100 μL of biotin-GOx (1 μg/mL) was added for another 0.5 h of reaction at 37 °C, after washing thrice with PBST and once with PBS to remove excess streptavidin. Free biotin-GOx was removed by washing, and a 1-hour incubation with 100 μL of glucose solution (1 mg/mL in PB, optimized pH described in Section 2.2) at 37 °C was performed. Lastly, 50 μL of MPA-QDs (diluted in PB, optimal concentration and pH value according to Section 2.2) was added for a 15-minute incubation at room temperature. Then, Em_590_ was recorded using a multimode microplate reader under a 310-nanometer excitation.

The concentrations of anti-OTA ascitic fluids and bifunctional phage in the FLISA procedure were optimized via a checkerboard titration method. Four × four assays with different parameters were conducted and compared according to the resultant fluorescence intensities of the OTA-negative (PBS, pH = 7.4) and OTA-positive samples (1 ng/mL in PBS buffer). Then, the competitive inhibition rates of the OTA-positive sample based on different conditions were calculated according to the following formula: (1 − B/B_0_) × 100%, where B and B_0_ are the fluorescence quenching rates of the OTA-negative and positive samples, respectively.

Under optimal concentrations of anti-OTA ascitic fluids and bifunctional phage, several key parameters of the sample solution, including the ionic strength, pH and methanol content were optimized in sequence. FLISA assays were performed under different conditions for detecting both OTA-negative (PBS) and OTA-positive samples (1 ng/mL in PBS). The resultant competitive inhibition rate of 1 ng/mL of OTA was chosen as the key quota for determining the optimal values of the NaCl concentration, pH and methanol content in sample solution. The biotinylated M13_OTA_ phage was diluted using PBS buffer with the same NaCl concentration and pH as the sample solution.

### 2.7. Corn Sample Pretreatment

Corn samples (Grain Procurement Agency of Shandong Province, China) were verified to be OTA free via HPLC. All the samples were pre-ground and mixed well before use. Samples were extracted in accordance with our previously reported procedure [32]. Briefly, 5.0 g of pulverized corn sample was spiked with the desired amount of OTA, and then was extracted with 25 mL of methanol-H_2_O solution (80%:20%, *v/v*) for 20 min under vortex shaking. After spinning at 10,000 rpm for 5 min, the supernatant was stored at 4 °C for analysis.

## 3. Results and Discussion

### 3.1. Principle of the Proposed M13_OTA_-FLISA Method

**Figure 1:**
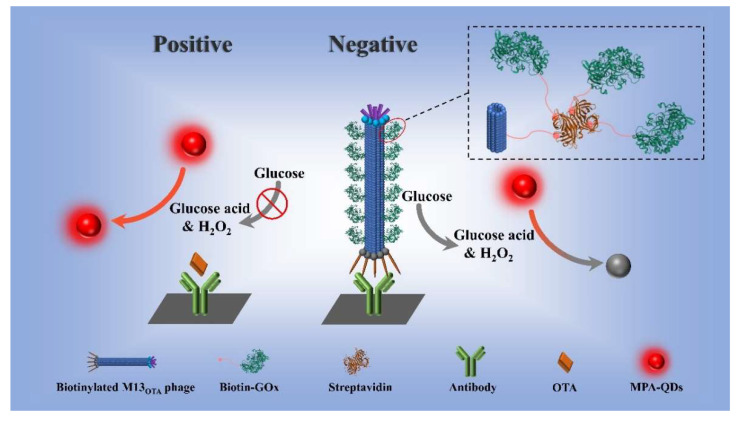
The principle of M13_OTA_-FLISA using bifunctional M13 phage as competing antigen and MPA-QDs as signal transducers.

Herein, the bifunctions of M13 phage as a competing antigen and enzyme container were integrated to establish an eco-friendly fluorescent ELISA (FLISA) for the highly sensitive detection of OTA in real corn. In detail, the p8 proteins of M13_OTA_ were functionalized with biotins; thus, they could be used for the high-density loading of GOx enzymes via the biotin–streptavidin system. Then, an M13_OTA_-FLISA was developed by using the biotinylated M13_OTA_ phage as an eco-friendly competing antigen and MPA-QDs as signal transducers (Figure 1). The biotinylated M13_OTA_ phage was captured by using an anti-OTA ascitic fluid-modified microplate in the absence of OTA. Then, the large amounts of GOx added later were captured, leading to the fluorescence quenching of MPA-CdTe QDs via H_2_O_2_ from the GOx-catalyzed oxidation of glucose. In the OTA-positive samples, the binding of M13_OTA_ and fluorescence quenching were inhibited via a competitive binding process.

### 3.2. GOx-Mediated Fluorescence Quenching of MPA-QDs

In the proposed FLISA, OTA was quantified on the basis of the GOx-mediated fluorescence quenching of MPA-QDs. MPA-capped CdTe QDs are sensitive to H_2_O_2_ that can be produced through GOx-catalyzed glucose oxidation. They also exhibit remarkable fluorescence quenching caused by thiolate detachment and tellurium oxidation. Herein, the fluorescence property of the used CdTe QDs was evaluated to select the optimal parameters of the signal transduction system. CdTe QDs exhibit the maximum emission peak at about 590 nm (Figure S2). The effect of pH on the fluorescence of the CdTe QDs was examined, given that fluorescence intensity is susceptible to the concentration of hydrogen ions in a solution (Figure S3). The results show that CdTe QDs have the highest fluorescence intensity at pH 7.5, whereas the fluorescence intensity drastically decreases when the pH decreases from 7.5 to 5.0. Then, the effect of QD concentration on the sensitivity to H_2_O_2_ was assessed. In theory, the lower the concentration of CdTe QDs is, the lesser the amount of H_2_O_2_ for fluorescence quenching will be. The results presented in Figure S4 show that the 12.5 nM QD solution is the most sensitive to H_2_O_2_, and its LOD for H_2_O_2_ is as low as 0.37 μM. However, the fluorescence intensity of 12.5 nM QDs is too low to provide a suitable signal variation in the presence of H_2_O_2_. Hence, the optimal QD concentration is 33 nM, with an LOD for H_2_O_2_ of 0.61 μM. GOx exhibits a high activity in a weakly acidic solution (pH = 5.0–7.0). Conversely, the fluorescence intensity of MPA-QDs is quenched in an acidic environment. Afterward, the GOx-induced fluorescence quenching of CdTe assays was conducted at different initial pH values in the presence of glucose (1 mg/mL) to explore the optimal pH that could favor the balance between the GOx activity and fluorescence intensity of QDs. The concentration of GOx leading to a 10% fluorescence quenching rate at pH 7.0 (11 pg/mL) is lower than those at pH 6.5 and 7.5 (Figure S5). Thus, the optimal pH of glucose solution and CdTe QD solution is 7.0, and the LOD for GOx under this condition is 4.6 pg/mL.

### 3.3. Characterization of Bifunctional M13_OTA_ Phage

**Figure 2:**
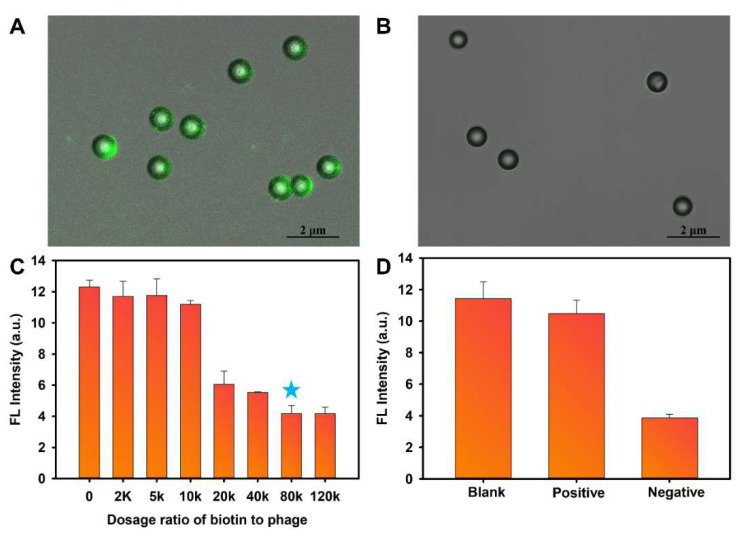
Characterization of biotinylated M13_OTA_ phage. (**A**) The confocal laser microscope images of streptavidin-polystyrene microspheres after incubating with streptavidin-FITC and biotinylated M13_OTA_ phage; (**B**) The confocal laser microscope images of streptavidin-polystyrene microspheres after incubating with streptavidin-FITC and unmodified M13_OTA_ phage; (**C**) Optimization of biotin to phage dosage ratio for preparing phage with biggest loading capacity. (**D**) Feasibility to apply the biotinylated M13_OTA_ phage as competing antigen in the FLISA for OTA detection. The blank group was conducted in the absence of the phage, the negative and positive groups were conducted with OTA negative and OTA positive (1 ng/mL) samples in the presence of biotinylated M13_OTA_ phage. The error bars represent the standard deviation of the three measurements and the blue star means the optimized conditions.

The M13_OTA_ phage with a peptide sequence “GMSWMMA” fused with the end of the p3 protein of the M13 phage [31] was modified with biotin molecules on its p8 proteins. To verify the avidin-specific binding ability of the resultant biotinylated M13_OTA_ phage, it was used as a bridge for conjugating streptavidin-modified polystyrene (PS) microspheres with streptavidin-FITC. Laser confocal microscopy reveals a bright green fluorescence on the surface of the microsphere (Figure 2A). Conversely, unmodified M13_OTA_ phage cannot form a bridge between streptavidin-FITC and the microsphere (Figure 2B). Thus, the as-prepared biotinylated M13_OTA_ phage can achieve high-density enzyme loading through the biotin–avidin system. Biotinylation was optimized to maximize the loading capacity of the M13_OTA_ phage. Immunoassays were conducted to develop “antibody–phage–GOx” sandwich structures on anti-OTA ascitic fluid-modified microplates, and the fluorescence intensity of the subsequently added CdTe QDs were detected to evaluate the performance of biotinylated M13_OTA_ phage. The results in Figure 2C demonstrate that the fluorescence quenching rate increases as the biotin-to-phage dosage ratio in the phage increases and reaches a plateau at a dosage ratio of 80,000:1. Therefore, biotinylated M13_OTA_ phage developed at the ratio of 80,000:1 saturates the loading capacity of the phage. Then, the loading capacity of streptavidin was evaluated in terms of the fluorescence difference between the devoted streptavidin-FITC in the phage solution and the unbonded streptavidin-FITC in the supernatant after centrifugation. The results illustrated in Figure S6 reveal that each biotinylated phage can bind to approximately 269 of streptavidin molecules, indicating the high loading capacity of biotin-GOx for signal amplification. Then, the performance of the bifunctional phage acting as a competing antigen in the proposed FLISA was assessed by parallelly conducting three assays (Figure 2D). In the blank control assay, no GOx was captured on the microplates because of the absence of the bifunctional phage, thus leading to an unchanged fluorescence intensity of CdTe QDs. Conversely, the resultant fluorescence intensity of CdTe QDs obtained from the detected OTA-negative sample in the presence of the bifunctional phage decreases considerably, accounting for the development of antibody–phage–GOx complexes on microplates. By contrast, the fluorescence intensity from the OTA-positive sample (1 ng/mL) is only slightly lower than that of the blank group, revealing that the formation of antibody–phage–GOx complexes is efficiently inhibited. These results demonstrate that the bifunctional phage can bind to the anti-OTA antibody, but this combination can be competitively inhibited by OTA. In conclusion, the proposed biotinylated M13_OTA_ phage can act as an eco-friendly competing antigen for OTA and serve as container for GOx enzymes to amplify output signals.

### 3.4. Development of the M13_OTA_-FLISA

A competitive FLISA involving phage as a competing antigen and a GOx container was developed on the basis of the excellent bifunction of biotinylated M13_OTA_ phage. Several key parameters were optimized to achieve the highest sensing sensitivity for OTA. First, the concentrations of anti-OTA ascitic fluids and bifunctional phage described in the FLISA procedure were optimized via the checkerboard titration method. In Table S1, the highest inhibition rate of 1 ng/mL of OTA is 83.57% when 1.5 μg/mL of anti-OTA ascitic fluids and 2 × 10^9^ pfu/mL of biotinylated M13_OTA_ phage were used in the assay procedure.

**Figure 3:**
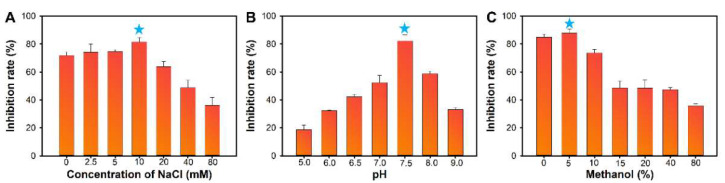
Optimization of the experimental conditions for the M13OTA-FLISA. Effect of (**A**) NaCl concentration (0~80 mM), (**B**) pH values (6.5–8.0), (**C**) methanol content (*v*/*v*, 0–80%) on the performance of the M13OTA-FLISA. The error bars represent the standard deviation of the three measurements and the blue star means the optimized conditions.

The ionic strength, pH and methanol content of the sample solution that could influence the immuno-recognition interactions were optimized on the basis of the resultant competitive inhibition rates of 1 ng/mL of OTA. Figure 3A shows that the competitive inhibition rate increases when the NaCl concentration in the sample solution increases from 0 to 10 mM. When >10 mM NaCl is added to the sample solution, the competitive inhibition rate decreases obviously. Hence, 10 mM is the appropriate NaCl concentration in the sample solution. Figure 3B presents the resultant inhibition rates obtained from detecting 1 ng/mL of OTA with different sample pH values. The results demonstrate that pH 7.5 favors the improvement of the assay sensitivity, leading to the highest inhibition rate. The extract solution containing a certain concentration of methanol is required because of the hydrophobic property of OTA molecule to ensure high extraction recovery from real samples. However, methanol can interfere with the interaction between antigens and antibodies. Thus, the effect of methanol concentrations of the immunoassay was investigated. As revealed in Figure 3C, no obvious change in the competitive inhibition rate is observed when the methanol content in the sample solution is less than 5%, whereas the competitive inhibition rate sharply decreases in the sample with a higher methanol concentration. In conclusion, the appropriate buffer for the OTA standard sample preparation is 10 mM PBS buffer (pH = 7.5) containing 10 mM NaCl and 5% methanol. For real sample preparation, the extract solution should be diluted with 10 mM PBS buffer (pH = 7.5) containing 10 mM NaCl until the final methanol content reaches 5%.

### 3.5. Analytical Performance of the M13_OTA_-FLISA

**Figure 4:**
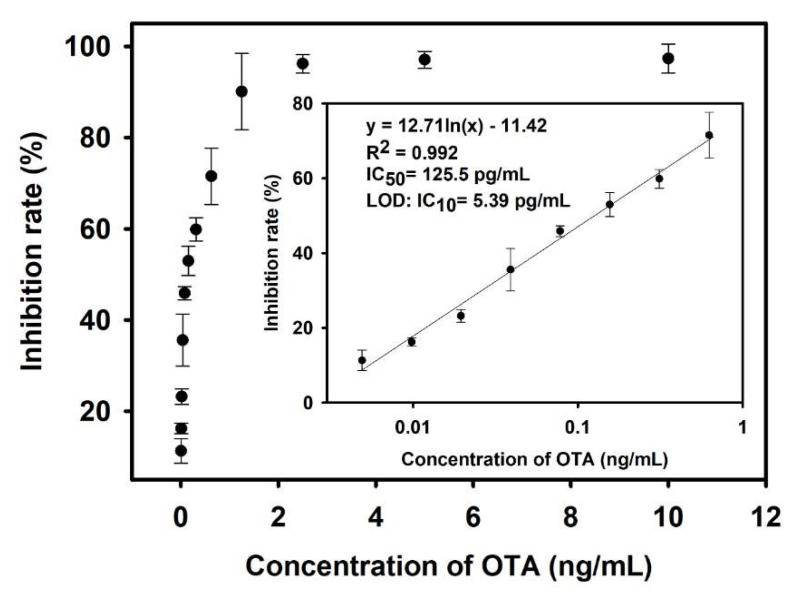
Competitive inhibition curve of the M13_OTA_-FLISA, the inset shows a dynamic linear range of OTA concentrations from 4.8 to 625 pg/mL. Each independent experiment was repeated 3 times. Error bars were based on triplicate measurements.

Under the optimized conditions, OTA standard solutions (0.001–10 ng/mL) were detected using the developed M13_OTA_-FLISA; therefore, a standard inhibition curve was established by plotting the resultant competitive inhibition rates against the OTA concentrations. Figure 4 shows that the standard curve displays a good linear relationship when the OTA concentration ranges from 4.8 to 625 pg/mL, and the corresponding regression equation can be *y* = 0.127 ln(*x*) − 0.1142 (R^2^ = 0.992). IC_50_ and 10% competitive inhibition concentration (IC_10_, equal to LOD) are calculated to be 125.5 and 5.39 pg/mL, respectively, indicating the high detection sensitivity of the M13_OTA_-FLISA. The LOD of the proposed M13_OTA_-FLISA is about 26-fold lower than that of a conventional HRP-based ELISA (0.14 ng/mL, Figure S7) and six-fold lower than that of the FLISA when GOx-OTA conjugates are used as the competing antigens (29.71 pg/mL, Figure S8). These results conclude that the biotinylated M13_OTA_ phage is a promising alternative competing antigen for an OTA-sensing immunoassay. It is harmless and can significantly improve detection sensitivity.

**Figure 5:**
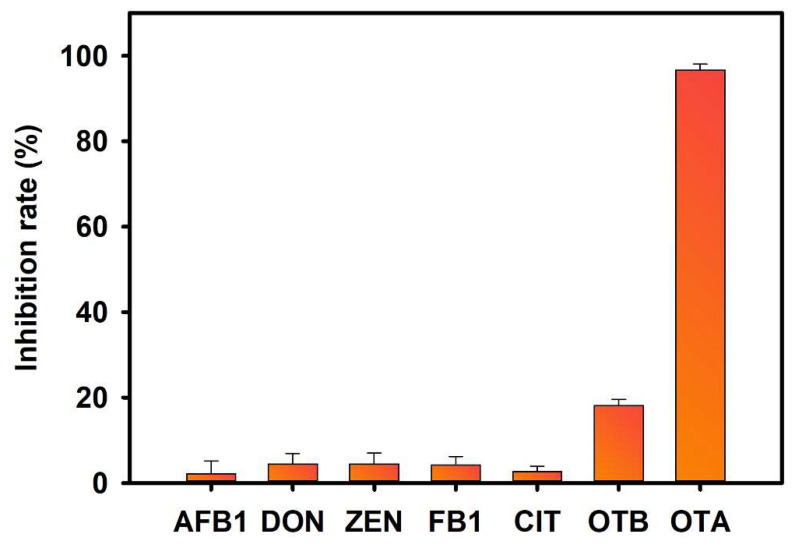
Cross-reactivity of the proposed M13_OTA_-FLISA. The samples were AFB1 (100 ng/mL), DON (100 ng/mL), ZEN (100 ng/mL), FB1 (100 ng/mL), CIT (100 ng/mL), OTB (1 ng/mL) and OTA (1 ng/mL). The error bars represent the standard deviation of the three measurements.

The specificity of M13_OTA_-FLISA was assessed by detecting several other mycotoxins at high concentrations, including AFB1 (1000 ng/mL), DON (100 ng/mL), ZEN (100 ng/mL), FB1(100 ng/mL), CIT (100 ng/mL), OTB (1 ng/mL) and OTA (1 ng/mL). Figure 5 shows that about a 20% inhibition rate is observed in the presence of OTB (1 ng/mL), whereas the samples containing five other toxic substances have an ultralow inhibition ability against the binding of the phage. Then, a standard inhibition curve of OTB is displayed in Figure S9, and the cross-reactivity for OTB is calculated to be 2.47% according to the following formula: CR = [IC_50_ for OTA]/[IC_50_ for OTB] × 100%.

The accuracy and precision of the M13_OTA_-FLISA were evaluated with recovery assays performed for corn samples spiked with OTA at different concentrations (2–160 μg/kg). The results displayed in Table 1 show that the average recoveries range from 90.16 to 116.45%, and the relative standard derivation is smaller than 15%. This result demonstrates the good accuracy and high reproducibility of the developed M13_OTA_-FLISA. Then, 15 OTA-spiked real corn samples were blindly analyzed through the ultra-performance liquid chromatography-fluorescence detection (UPLC-FLD) method and our proposed method to validate the reliability and practicability of our proposed ELISA. Table 2 shows that the detection results of our proposed method are similar to those of the UPLC-FLD method. Moreover, the two methods show a highly significant correlation (R^2^ = 0.9677; Figure S10). Therefore, the proposed method can be used for the accurate and reliable detection of OTA in real corn samples.

## 4. Conclusions

In summary, a novel FLISA method for OTA was developed using a biotinylated M13_OTA_ phage as a green competing antigen and H_2_O_2_-sensitive MPA-QDs for signal transduction. The biotinylated M13_OTA_ phage was demonstrated to be a good OTA mimicking the competing antigen that could promote analytical sensitivity by acting as a container for the loading of a large amount of GOx. The proposed M13_OTA_-FLISA showed a high sensitivity for OTA detection with an LOD of 5.39 pg/mL, which was 26-fold lower than that of conventional HRP-based ELISA and six-fold lower than that of a GOx-OTA conjugate-based FLISA. UPLC-FLD validated that our proposed method had high accuracy and precision. As various mimotopes, ligands and nanobodies against different target analytes could be panned from phage display libraries. The proposed strategy also showed potential for the highly sensitive detection of other small organic molecules, macromolecules and even pathogens.

## Figures and Tables

**Table 1:** Recoveries of OTA-spiked corn samples.

Spiked-OTA (μg/kg)	Intra-Assay (n = 3)			Inter-Assay (n = 3)		
	OTA Recovered (μg/kg)	Recovery (%)	CV (%)	OTA Recovered (μg/kg)	Recovery (%)	CV (%)
2	2.06 ± 0.32	116.45	13.86	2.13 ± 0.19	106.74	8.83
8	8.11 ± 0.62	101.32	7.71	7.36 ± 0.96	92.02	13.06
40	41.12 ± 4.78	102.79	11.62	38.64 ± 4.03	96.59	10.44
80	84.76 ± 6.96	105.95	8.21	80.89 ± 6.47	101.12	8.04
120	108.2 ± 16.02	90.16	14.81	115.51 ± 11.32	96.26	9.80
160	159.93 ± 19.67	99.96	12.30	155.41 ± 14.92	97.13	9.60

**Table 2:** Comparison of results obtained from detecting real corn samples using the proposed M13_OTA_-FLISA and UPLC-FLD method.

Incurred Samples	M13_OTA_-FLISA	UPLC-FLD
	OTA Recovered (μg/kg)	OTA Recovered (μg/kg)
1	103.26 ± 3.12	119.91
2	42.60 ± 5.41	33.79
3	25.41 ± 3.86	21.49
4	40.53 ± 3.49	52.60
5	159.74 ± 21.31	163.76
6	57.48 ± 0.63	76.06
7	28.75 ± 1.29	31.07
8	183.68 ± 9.38	208.76
9	94.56 ± 1.53	88.76
10	21.72 ± 1.6	19.14
11	16.79 ± 0.84	16.83
12	53.18 ± 0.71	61.83
13	132.76 ± 9.33	144.91
14	26.07 ± 0.81	25.80
15	37.21 ± 2.84	34.53

## Data Availability

The data presented in this study are available on request from the corresponding author (Y.L.), upon reasonable request.

## Supplementary Materials

The following are available online at https://www.mdpi.com/article/10.3390/foods10102429/s1, Figure S1: M13 bacteriophage titer determination, Figure S2: Photoluminescence and UV-vis absorption spectra of CdTe QDs, Figure S3: Effect of pH value on fluorescence intensity of MPA-QD, Figure S4: Fluorescence quenching of MPA-QDs induced by H_2_O_2_ at different QD concentrations, Figure S5: Fluorescence quenching rate of MPA-QDs induced by GOx in the presence of glucose at different pH values, Figure S6: Binding capacity of biotinylated M13_OTA_ phage for streptavidin, Figure S7: Calibration curve of conventional HRP-based ELISA, Figure S8: Calibration curve of FLISA using OTA-GOx conjugates as competing antigens and MPA-QDs as signal transducers, Figure S10: Comparison of results obtained from detecting real corn samples using the proposed M13_OTA_-FLISA and UPLC-FLD method, Table S1: Optimization of the working conditions of coating antibody and biotinylated M13_OTA_ phage using checkerboard method.
