# Supplementary material for: Eco-Friendly Fluorescent ELISA Based on Bifunctional Phage for Ultrasensitive Detection of Ochratoxin A in Corn
Source: Foods. 2021 Oct 13;10(10):2429. doi: 10.3390/foods10102429 (PMC8536128; doi:10.3390/foods10102429)
Supplement: Supplementary file 1 [file foods-10-02429-s001.zip › foods-1399010-supplementary.pdf]

Supporting Information

Eco-friendly fluorescent ELISA based on bifunctional phage for ultrasensitive detection of Ochratoxin A in corn

Weipeng Tong [1,2], Hao Fang [1,2], Hanpeng Xiong [1,2], Daixian Wei [1,2], Yuankui Leng [1,2,*], Xinyu Hu [3], Xiaolin Huang [1,2] and Yonghua Xiong [1,2,4]

1 State Key Laboratory of Food Science and Technology, Nanchang University, Nanchang 330047, China; 402337519017@email.ncu.edu.cn (W.T.); 357900210001@email.ncu.edu.cn (H.F.); 402337520004@email.ncu.edu.cn (H.X.); 412314919055@email.ncu.edu.cn (D.W.); xiaolin.huang@ncu.edu.cn (X.H.); yhxiongchen@163.com (Y.X.)

2 School of Food Science and Technology, Nanchang University, Nanchang 330047, China

3 School of Qianhu, Nanchang University, Nanchang 330031, China; 7901119026@email.ncu.edu.cn

4 Jiangxi-OAI Joint Research Institute, Nanchang University, Nanchang 330047, China

* Correspondence: ykleng@ncu.edu.cn

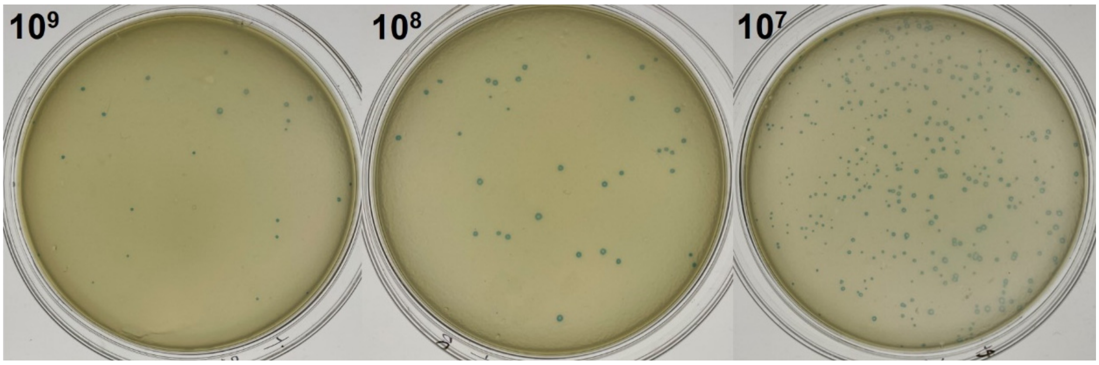

Figure S1. M13 bacteriophage titer determination plate physical map performed with 10 μL of phage solutions diluted with 10^7 to 10^9-folds.

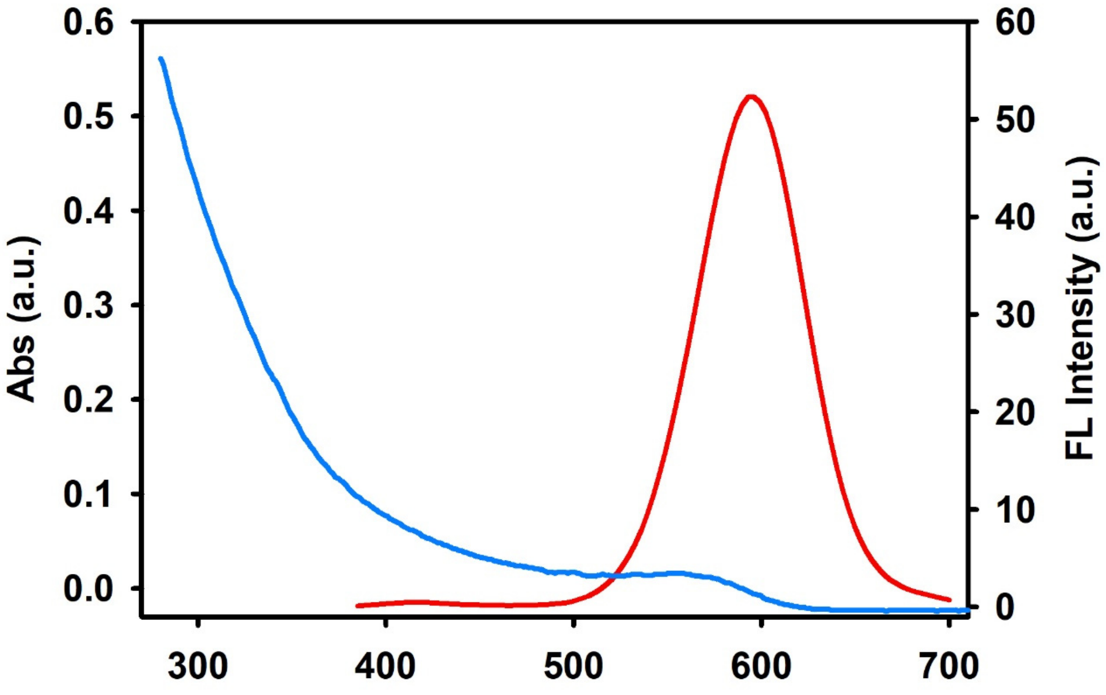

Figure S2. Photoluminescence and UV-vis absorption spectra of CdTe QDs.

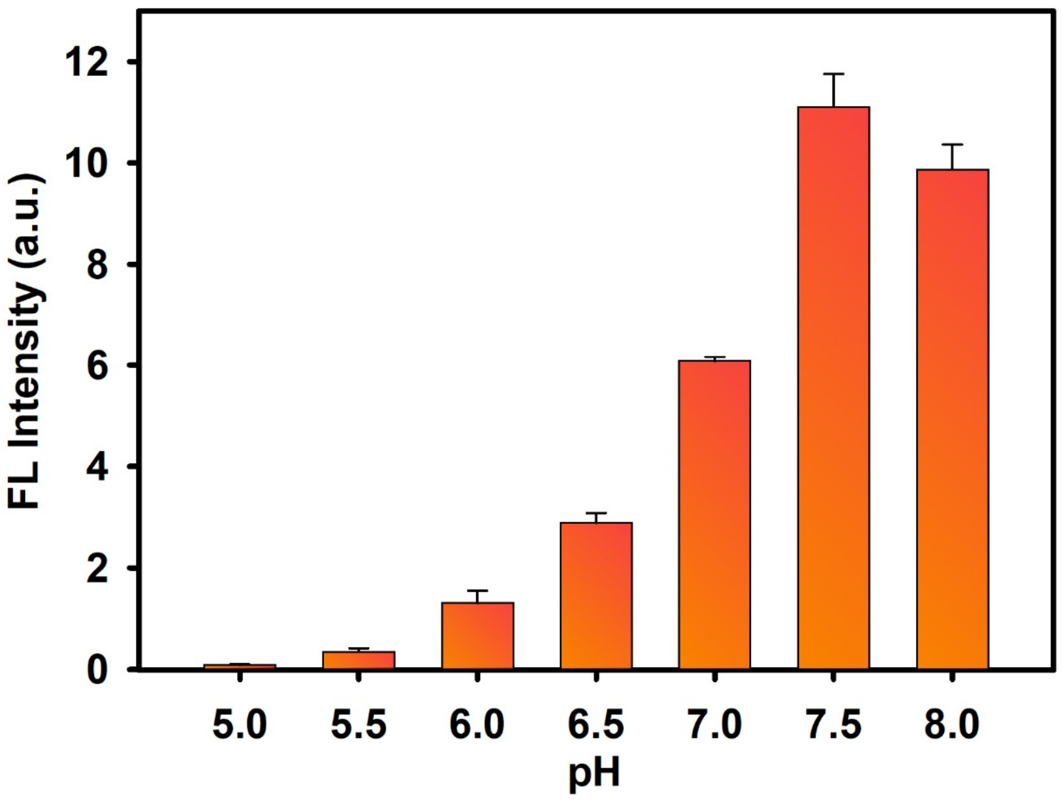

Figure S3. Effect of pH value on fluorescence intensity of MPA-QDs (25 nM). The error bars represent the standard deviation of the three measurements

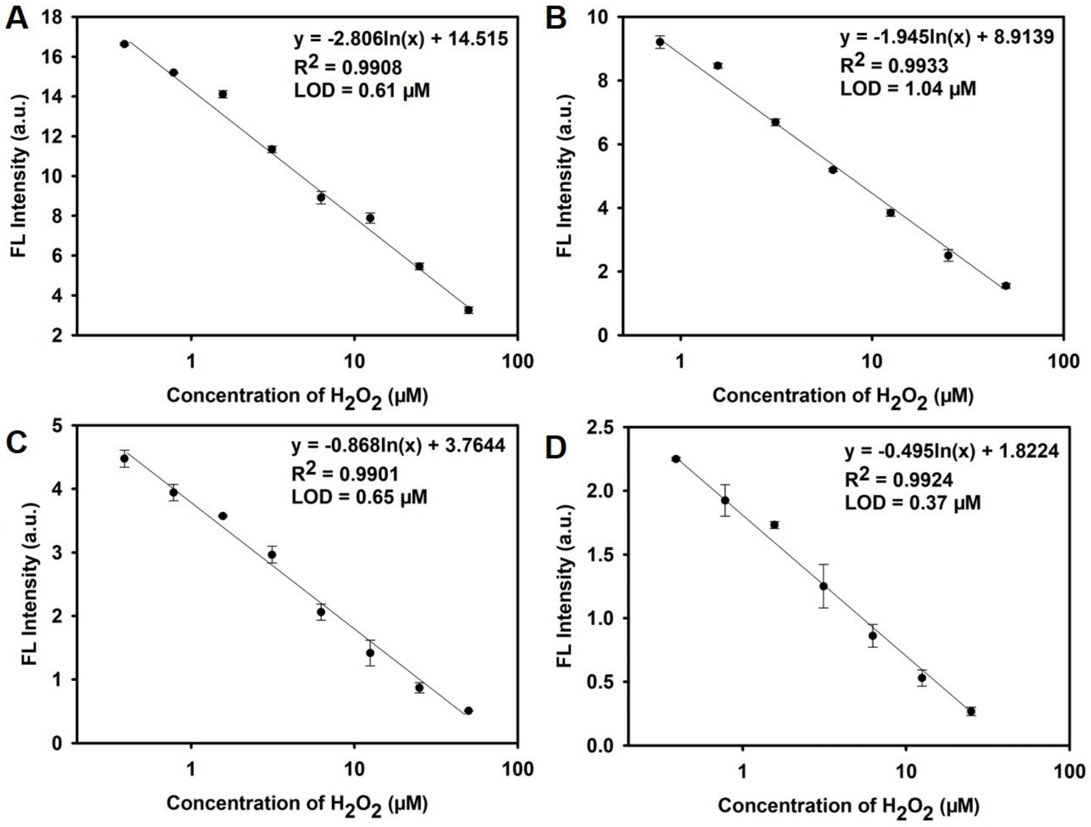

Figure S4: Fluorescence quenching of MPA-QDs induced by H_2O_2 at different QD concentrations (A) 33 nM; (B) 25 nM; (C) 16.5 nM; (D) 12.5 nM. The pH of this test was 7.5. The error bars represent the standard deviation of the three measurements

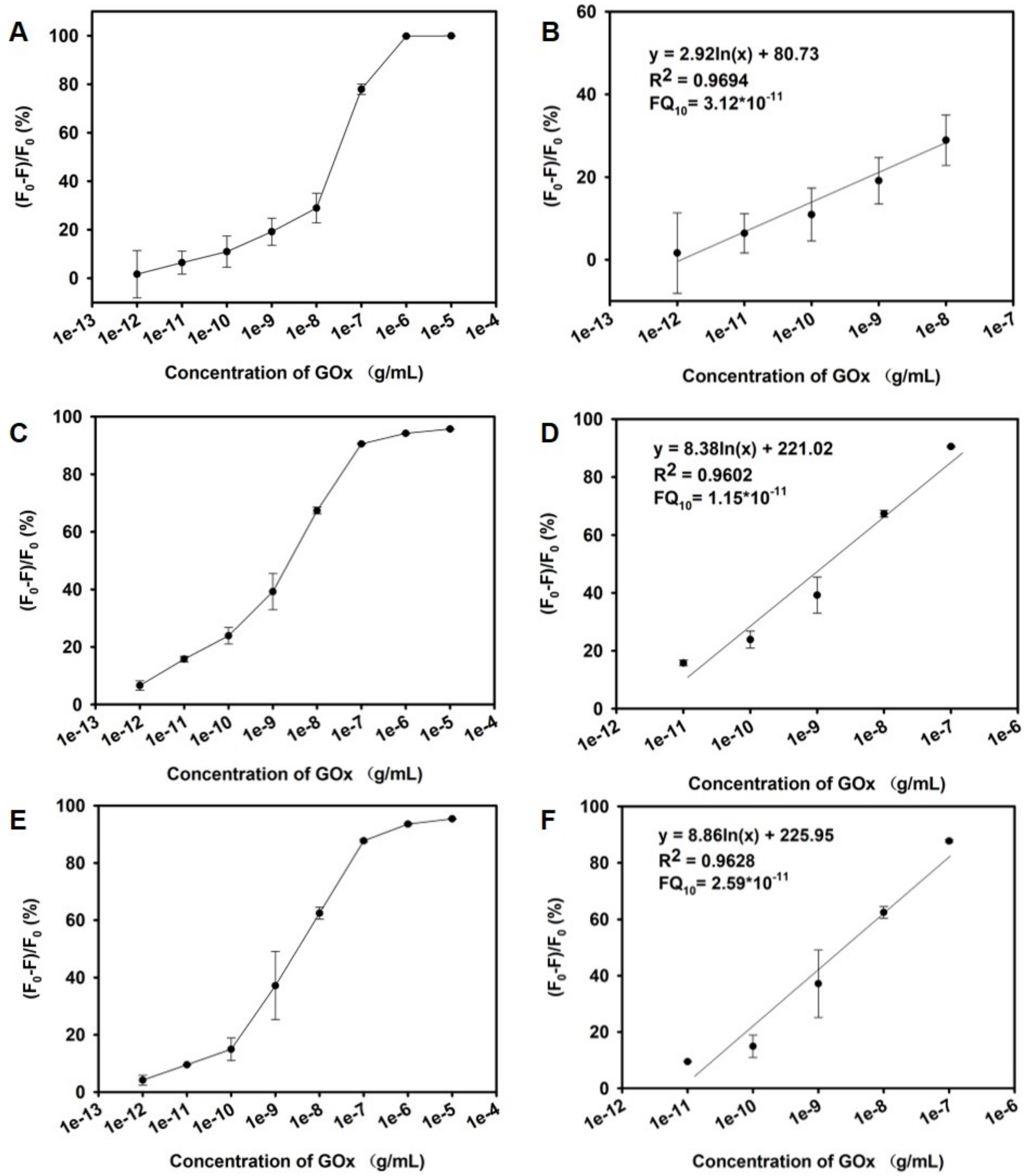

Figure S5. Fluorescence quenching rate of MPA-QDs (33 nM) induced by GOx in the presence of glucose at different pH values. (A-B) pH 6.5; (C-D) pH 7; (E-F) pH 7.5.

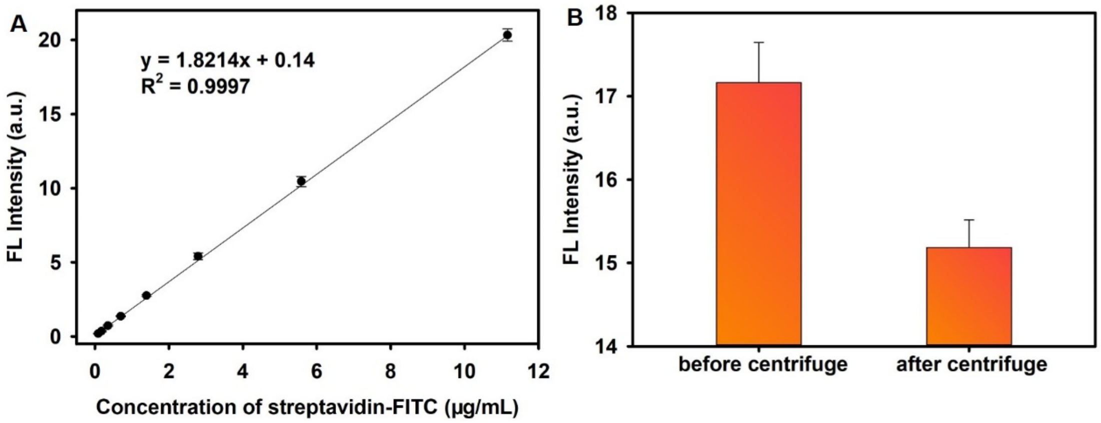

Figure S6. (A) Standard curve by plotting fluorescent intensity against the concentration of streptavidin-FITC. (B) Fluorescent intensity of the mixture of streptavidin-FITC and $3.7*10^{10}$ pfu/mL biotinylated phage before centrifugation and that in the supernatant after centrifugation. First, the concentration of streptavidin-FITC in the mixture is calculated to be 9.35 μg/mL, and the concentration of streptavidin-FITC in the supernatant is calculated to be 8.26 μg/mL. Then the loading capacity of the phage for streptavidin is calculated to be N=$\frac{(9.35-8.26)\ \mu g/mL}{3.7*10^{10} pfu/mL} * \frac{6.02*10^{23}\ /mole}{6.6*10^{4}\ g/mole} \approx 269$

`

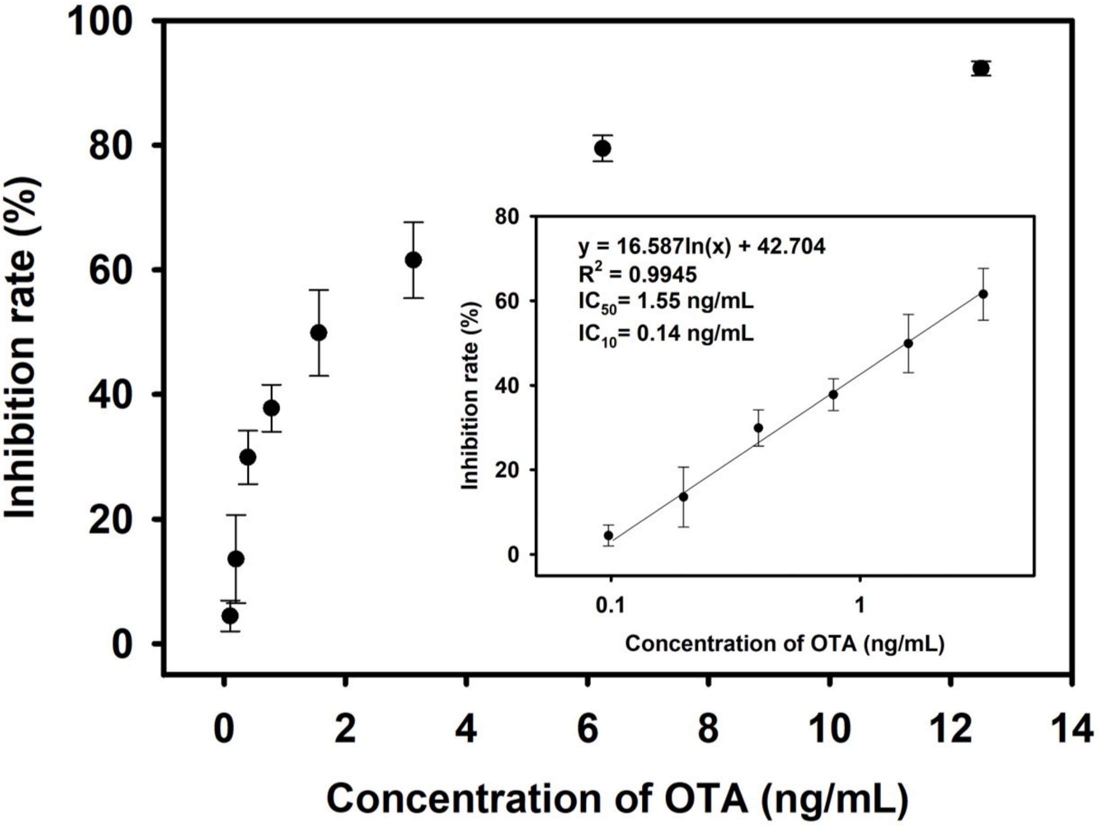

Figure S7. Calibration curve of conventional HRP-based ELISA, the inset shows a dynamic linear range of OTA concentrations from 0.098 ng/mL to 3.125 ng/mL. Each independent experiment was repeated 3 times.

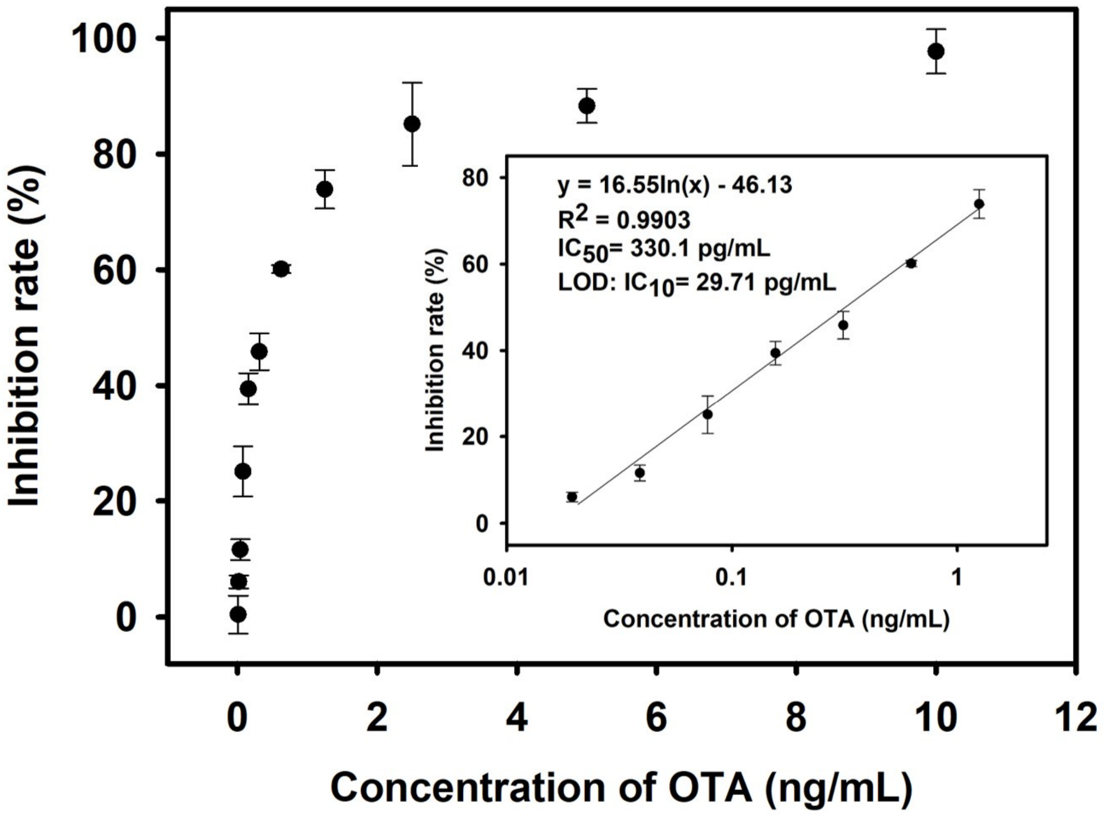

Figure S8. Calibration curve of FLISA using OTA-GOx conjugates as competing antigens and MPA-QDs as signal transducers, the inset shows a dynamic linear range of OTA concentrations from 19.5 pg/mL to 1250 pg/mL. Each independent experiment was repeated 3 times.

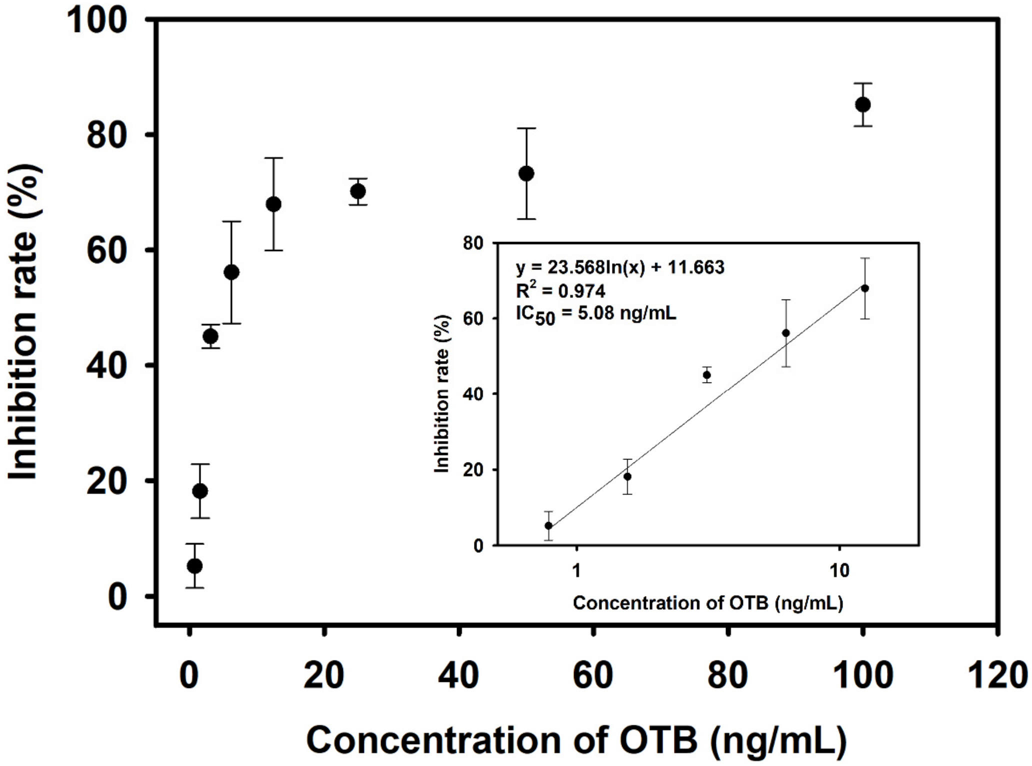

Figure S9. Calibration curve of the proposed $M13_{OTA}$-FLISA for OTB, the inset shows a dynamic linear range of OTB concentrations from 0.5 ng/mL to 12.5 ng/mL. Each independent experiment was repeated 3 times.

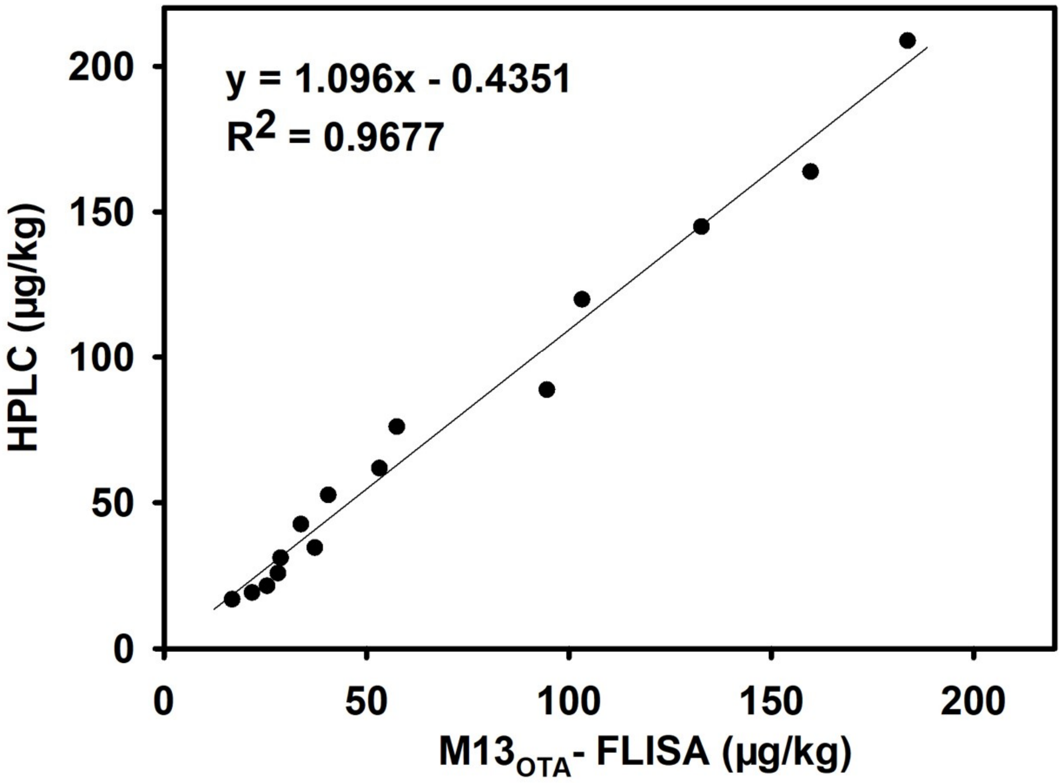

Figure S10. Comparison of results obtained from detecting real corn samples using the proposed $M13_{OTA}$-FLISA and UPLC-FLD method (n = 15).

Table S1. Optimization of the working conditions of coating antibody and biotinylated $M13_{OTA}$ phage using checkerboard method. The competitive inhibition rates: $(B_0-B)/B_0 \times 100\%$ was used to confirm the optimal parameters, where B_0 and B represented the fluorescent quenching rate of negative sample (OTA-free) and an OTA-spiked PBS solution (1 ng mL^{-1}), respectively.

Concentration of anti-OTA ascitic fluids (μg/mL)	Concentration of Bio-phage (pfu/mL)			
	4×10^9	2×10^9	1×10^9	5×10^8
3.00	41.72%	52.99%	46.04%	37.99%
1.50	55.13%	83.57%*	76.15%	59.53%
0.75	67.80%	77.19%	66.15%	47.46%
0.38	50.03%	55.71%	61.44%	46.53%

The optimal concentrations of anti-OTA ascitic fluids and biotinylated $M13_{OTA}$ phage are 1.5 μg/mL and 2×10^9 pfu/mL, respectively.
